# The Influence of Telehealth for Better Health Across Communities

**DOI:** 10.5888/pcd17.200254

**Published:** 2020-07-16

**Authors:** Jane A. McElroy, Tamara M. Day, Mirna Becevic

**Affiliations:** 1 Family and Community Medicine Department, University of Missouri, Columbia, Missouri; 2 University of Missouri Health Care, University of Missouri, Columbia, Missouri; 3 Department of Dermatology, University of Missouri, Columbia, Missouri

## Abstract

Rapid spread of coronavirus disease 2019 (COVID-19) forced an abrupt shift in the traditional US health care delivery model to meet the needs of patients, staff, and communities. Through federal policy changes on telehealth, patient care shifted from in-person to telephone or video visits, and health care providers reached out to patients most at risk for exacerbation of chronic disease symptoms. ECHO (Extension for Community Healthcare Outcomes), a videoconferencing peer learning application, engaged health care providers across Missouri in the treatment and management of complex COVID-19–positive patients. Re-envisioning health care in the digital age includes robust utilization of telehealth to enhance care for all.

**Summary**

**What is already known on this topic?**

The COVID-19 pandemic has forced many health care institutions to reconsider health care delivery mechanisms. Because of reimbursement restrictions, telehealth has been underutilized.

**What is added by this report?**

Capitalizing on existing infrastructure that supports digital connectivity through uptake of telehealth was vital to successfully reimagine safe health care delivery. Removing a critical barrier, reimbursement, supported telehealth.

**What are the implications for public health practice?**

Technologic advancements and policy changes can alter health care delivery and have the potential to reduce disparities in access to care and improve outcomes among the most vulnerable populations.

## Introduction

In December 2019, an infection caused by a bat-origin novel coronavirus, severe acute respiratory syndrome coronavirus 2 (SARS-CoV-2), was detected in Wuhan, China (1). Within less than 3 months, coronavirus disease 2019 (COVID-19), the disease caused by SARS-CoV-2, had spread across China and worldwide. The World Health Organization declared COVID-19 a pandemic on March 11, 2020 (2). As of May 30, 2020, more than 1 million infections had been laboratory-confirmed in the United States with more than 100,000 case fatalities (3). An estimated 80% of people infected with COVID-19 during this time did not require hospitalization, and approximately 5% to 12% of hospitalized patients were admitted to intensive care units (3). Hospitalization rates were highest among adults aged 65 years or older, people with multiple chronic conditions, and men (3). Among younger patients (18–49 y), obesity, underlying chronic lung disease (primarily asthma), and diabetes were the most prevalent chronic disorders (3). Because COVID-19 is a pandemic, the virus is expected to cause multiple waves of infection in future months and to persist to cause seasonal outbreaks (2).

Patients exhibiting severe symptoms related to COVID-19 were urged to seek immediate care; however, this was challenging for people in rural areas of the United States, who make up about 20% (60 million residents) of the total population (4). Rural populations in the United States face significant challenges in accessing health care and have poorer health outcomes than urban or suburban populations, including higher rates of chronic disease, higher death rates, and delayed diagnoses for cancers and other diseases (5–7). These challenges are likely due to less accessible care related to lower rates of insurance; maldistribution of the health care workforce, particularly specialists; an older population; a greater proportion of patients with multiple comorbidities; and higher levels of socioeconomic need (8).

Missouri is a predominantly rural state. More than 97% of its land area is classified as rural, and from 30% to 37% of its population currently live in rural areas (9,10). Enriquez et al reported that at least 50% of patients in their Missouri study had one or more chronic diseases, and that “patients with multiple chronic conditions were the norm” (11). These comorbid conditions among rural Missouri residents put them most at risk of fatal complications from COVID-19, in particular those with predisposing conditions, such as diabetes, chronic pulmonary disease, and hypertension (3). As cases of COVID-19 increased exponentially once the pandemic reached the United States, clinicians and researchers became particularly concerned about its impact on the most vulnerable rural and underserved people with chronic conditions. Our objective is to describe the multipronged approach used in Missouri to provide quick response to the COVID-19 pandemic along with preliminary trend data, including disruptive technology applications that created an environment for widespread adoption of telemedicine.

Taking advantage of the experiences of US coastal cities where the COVID-19 pandemic hit hard and fast, an incident command team was created on March 9, 2020, at a tertiary referral hospital system, University of Missouri Health Care (MU Health Care), serving a 25-county, predominantly rural, catchment area. The team was co-led by the hospital’s chief nursing officer and chief medical officer because each profession brought a unique perspective. Policies were rapidly implemented that greatly reduced or suspended medical and surgical services to reserve personal protective equipment, reduced the clinical staff’s COVID-19 exposure, limited the number of patients and visitors in hospital, re-deployed staff, and extensively expanded the telemedicine infrastructure.

In this commentary, we use telehealth as an umbrella term referring to telemedicine and other health-related virtual activities, such as distance continuing medical education, training, and patient portals. Telemedicine will refer to providing medical care at a distance, which includes audio–video care or audio care only.

## Workforce Redeployment

The MU Health Care system had to reconsider the delivery of care, not only for the expected deluge of COVID-19–positive patients but also the routinely sick patients. With the governor’s stay-at-home edict and fear of SARS-CoV-2 exposure, patients were reluctant to actively seek medical care or keep scheduled appointments. With these policies and behavior changes, a significant shift in nursing work duties and the way nurses provided care occurred, often in areas outside normal clinical specialty areas. In response, almost 50% of the 1,836 patient care staff completed online rapid acute care orientation within 2 weeks of implementation to competently take on pivotal changes in work responsibilities.

On March 19, 2020, 3 quick-care clinics located in grocery stores were closed to redeploy advanced practice nurses to triage hundreds of patients who were calling with reports of respiratory illness (n = 1,368 through March 27, 2020). This strategy effectively reduced the need for clinic or emergency department in-person visits while continuing to address patients’ health care needs. During this same time period, some redeployed nurses served as ambulatory care coordinators and identified patients most at risk for exacerbation of chronic disease symptoms. Coordinators initially contacted these vulnerable patients (n = 750) by telephone but transitioned eventually to audio–video consultations, when possible. Care coordinators checked in with patients regarding their health and well-being and closely collaborated with the patient’s primary care provider to coordinate any necessary medical care. This repositioning of nurses to care for vulnerable populations was based on strong evidence-based research in which nurse-led interventions in primary care have been shown to improve health outcomes (12). This also harkened back to an era in which patients stayed at home and the health care provider traveled to the patient. In this case, the traveling was virtual.

## Adoption of Telehealth

To readily support virtual traveling within the US health care system, the federal government allowed a more robust use of telehealth services during this national emergency. Specifically, the Centers for Medicare and Medicaid Services (CMS) made a limited-time change for allowable reimbursement for medical visits by expanding their definition to include telemedicine visits. The change was initially released on March 17, 2020, and made retroactive to March 6, 2020. CMS also relaxed the Health Insurance Portability and Accountability Act (HIPAA) requirements for secure exchange sites by allowing the use of nonpublic-facing video applications (such as Skype or Zoom) and text-based applications (such as WhatsApp, iMessage) (13). Within 24 hours of CMS’s decision to support telemedicine visits, our MU Health Care system had in place a structure to allow health care providers to use the technology for audio-visual visits. The ability of a large health care system to make this happen nearly overnight was breathtaking and a reminder of our potential to respond to an imminent challenge or threat. With this change, health care providers took care of both new and established patients in their homes by telephone and video visits (ie, telemedicine visits) throughout the 25-county catchment area.

Before the COVID-19 pandemic, reimbursement guidelines were an effective barrier to telemedicine use for both primary and specialty care with less than 1% of rural Americans using telehealth and few health care providers embracing it (14,15). In our MU Health Care system of selected specialties — family and community medicine, internal medicine, cardiology, and specialty medicine — no telehealth visits happened before March 2000 (Figure). Our visits peaked in April with almost 90% of visits happening through telehealth. With the lifting of the governor’s stay-at-home edict and opening of clinics, for the month of May the percentage of telehealth visits and the percentage of cancelled appointments reverted to March levels (Figure). In reviewing the 2019 appointment data, May had a higher volume of appointments than February through April. An opposite pattern for the same time period was observed in 2020; May had the lowest volume of appointments. This leads us to conclude that the appointment trends we are observing are not associated with seasonality. We attribute a lower number of appointments in May and higher number of cancellations to the continued public health response to the COVID-19 pandemic. Unfortunately, appointment cancellation data were not collected on type of visit so we do not have insight into whether telehealth versus in-person visits were more likely to be cancelled.

**Figure:**
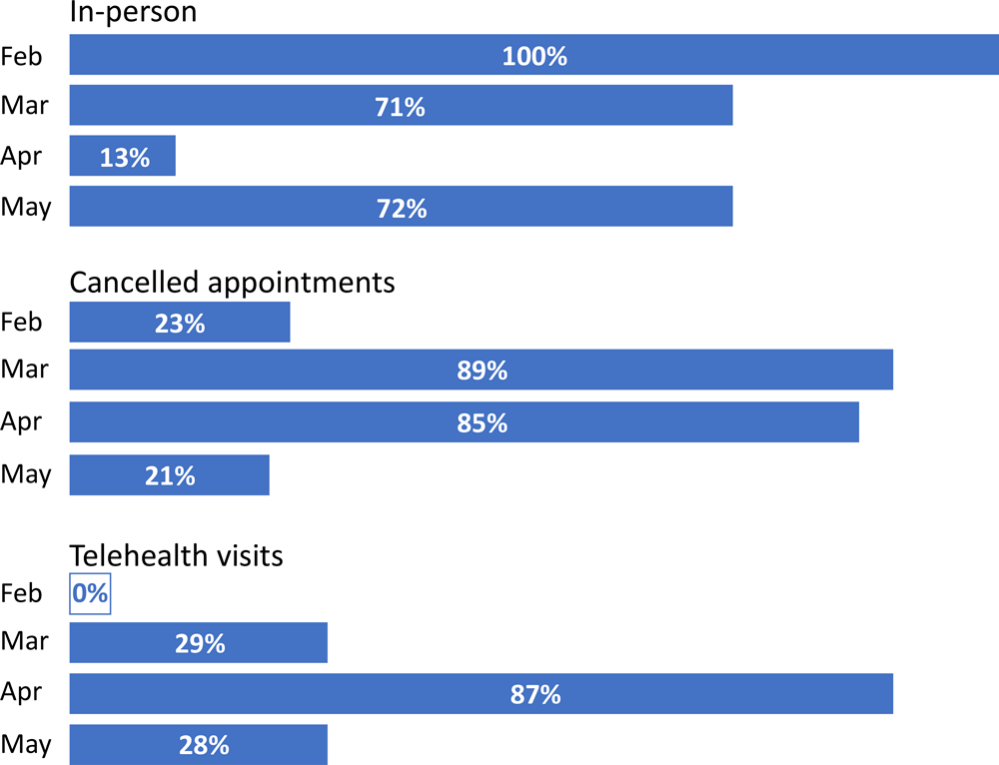
Percentage of ambulatory patients who had in-person clinical, cancelled, and telehealth visits for family medicine, internal medicine, cardiology, and medical specialty, February­–May, 2020. The denominator for in-person visits and telehealth visits is in-person plus telehealth visits. The denominator for cancelled appointments is all visits plus cancelled visits.

**Table:** 

Status of Clinic Visit, %	February	March	April	May
In-person visits	100	71	13	72
Cancelled appointments	23	89	85	21
Telehealth visits	0	29	87	28

For connectivity, another rapid change was CMS aligning audio-only with audio–video telemedicine care on April 30, 2020, retroactive to March 6, 2020. Originally audio-only visits were reimbursed at about one-third the rate of audio–video visits (16). For patients who experienced poor connectivity, this disparity in reimbursement had the potential to affect care and widen the gap in medical care for vulnerable populations. Missouri is ranked fortieth among states on the digital divide index; this score is derived by using both broadband access and broadband adoption as well as socioeconomic factors (17). The presence of any broadband in households within our 25-county service area ranges from 60% to 82%. The literature on the telehealth divide between rural and urban areas shows that the quality of broadband access affects the use of telehealth (18,19), although some disparities are narrowing (18). Besides connectivity, other factors influence the ability to use telehealth. In preliminary data using family medicine encounters over a 1-month period (March 17–Apr 16, 2020), telemedicine services with audio–video visits were markedly less likely to be among older, black, Medicaid-insured, or self-pay patients. Schmeida and McNeal found that among demographic groups less likely to have internet access at home, including the poor, older patients, Latinos, and Blacks, limited internet access could affect the way they used the internet for telehealth and/or searched for health care–related information online (18).

Our MU Health Care system sent a patient experience survey to all who had a telehealth visit from March 19, 2020, to June 3, 2020 (N = 4,183), and 25% responded. Half were asked the question, “Was your telehealth visit as good as an in-person appointment?” to which 33% gave a positive response. The other half were asked, “Was it easy to state concerns and ask questions through telehealth?” to which 56% gave a positive response. Going forward, re-envisioning health care in the digital age in which health care providers are reimbursed for time spent with the patient virtually shifts the driver from insurance companies to patients and health care providers to determine what a health care visit looks like and to document access disparities, such as connectivity, privacy, and digital literacy. Therefore, more immediate and urgent action is needed to address these disparities for equitable health care in the adoption of telehealth.

## Virtual Collaborative Learning Network

Beyond the local response to the pandemic, a statewide response (Show-Me ECHO) was initiated by using Missouri Telehealth Network’s Extension for Community Healthcare Outcomes infrastructure. The Show-Me ECHO uses disruptive innovation technologies, such as videoconferencing applications, and is different from traditional telehealth. It is centered on case-based learning, health care provider development and retention, and efficiency. Although successfully adopted in acute care medicine and nonmedical applications, this model is primarily used to increase capacity of health care providers to care for patients with chronic diseases and targets rural, isolated, and underserved communities (20). Since its inception in Missouri, over 27,000 learners (medical doctors, doctors of osteopathy, nurse practitioners, physician assistants, health educators, and others) have attended sessions representing almost every county in Missouri. The existing infrastructure of this provider-facing technology was immediately expanded to create 2 new ECHOs: COVID-19 ECHO and Telemedicine ECHO.

COVID-19 ECHO, launched on March 23, 2020, supports health care professionals, especially those practicing in rural and isolated areas, with weekly meetings of didactic presentations focused on testing, triage, and other State updates, with more than 2,700 attendees as of May 26, 2020. De-identified case presentations were used for learning through a guided practice model, focusing specifically on patients with chronic conditions and COVID-19 infection or risk of infection. In addition to these weekly sessions, COVID-19–related topics were incorporated into other regular ECHO sessions, such as asthma, kidney disease, autism, and oral health, thereby substantially expanding the learning and networking opportunities among health care providers. A benefit of ECHO learning is the development of a network of professional colleagues that encourages informal communication outside of regular sessions. The spread of COVID-19 has caused fear and uncertainty among the public and concerns among health care professionals about their responsibilities to practice medicine while balancing their need to protect their families. The ECHO virtual collaborative network provides an ideal environment for reducing a sense of isolation among rural health care providers.

To support a growing number of novice health care providers using telemedicine and in response to popular demand, Telemedicine ECHO was initiated on April 14, 2020. Telemedicine ECHO is a collaboration of the University of Missouri, Missouri Telehealth Network, and the Heartland Telehealth Resource Center serving Missouri, Kansas, and Oklahoma. Telemedicine ECHO has provided didactic presentations on numerous topics, such as legal and regulatory issues, policy changes, billing and reimbursement, privacy, and security. The program has had more than 300 attendees as of May 26, 2020. Case presentations of patients with acute and chronic conditions included best practices for treatment and care management using telemedicine. Although many institutions, nudged by the COVID-19 pandemic, have adopted this technology, there is still an art to this type of encounter. As Telemedicine ECHO demonstrated, practicing health care professionals benefited from expert telemedicine support. It is likely that medical schools and residency programs will supplement their curricula on patient encounters to include telehealth visits, if it is not already included. As telemedicine becomes more commonly used, this platform can be extended to monitor those with influenza-like illness and COVID-19–like symptoms as well as assist in the management of multiple chronic diseases, as demonstrated by our Italian colleagues (21).

## Implications for Public Health

The success of MU Health Care’s rapid adjustment and response to the COVID-19 pandemic lies in its dedicated workforce, strong collaborative learning network, expertise in rural health, and robust telehealth infrastructure. One comment made by leadership on the COVID-19 response team was the unwavering willingness of nurses and other health care workers to go where they were needed. This especially epitomized the dedication and professionalism of nurses and health care professionals. Innumerable stories in the media abound of nurses filling in gaps created by new policies, such as restriction on visitors to hospitalized patients and being that hospitalized patient’s touchstone. As the COVID-19 first wave passes, the health care workforce, including nurses, can continue using telehealth successfully, and its use has been extended to departments and specialties that had never implemented telehealth before the pandemic. One of our gynecologic oncologists began using telemedicine after COVID-19 policies were enacted. He remarked that he plans on continuing telemedicine encounters for enhanced patient-centered care and that telemedicine provided more comprehensive family engagement. All family members participated in a telemedicine visit, asked questions, heard his responses, and understood the treatment plan and prognosis. Our oncologist felt the telemedicine encounter allowed the extended family to actively participate in the patient’s cancer journey. Without COVID-19’s disruption of the status quo of health care, it is unlikely that this example of re-envisioning the practice of health care would have occurred.

The pattern of delivering health care continues to adapt to medical, economic, and cultural changes. Before the middle of the twentieth century, few hospitals existed, and the health system enterprise, including health insurance, was nonexistent (22). Doctors traveled to their sick patients’ homes, provided limited treatment options, and were paid a modest out-of-pocket fee. Pivotal advances in scientific medical knowledge dramatically changed the landscape of medicine. The evolution from health care providers as generalists who provided all care for their patients to health care providers who refer their patients to specialists is complicated, but most consider that the tipping point in this change began in the post-World War II era (23). Currently, approximately 30% of younger patients (≤64 y) are referred to specialty care, and among older patients (≥65 y), referral to specialists average 2 per person per year (24). In the initial response to the COVID-19 pandemic, limited referrals for specialty care as well as appointment cancellations by health care providers for established patients and patients opting to not seek routine care were the norm, leaving a group of patients temporarily adrift (Figure). Similarly, just as technology, such as the invention of the telephone and automobiles, shaped health care by reversing the traveler — the patient coming to see the physician rather than physician going to the patient — disruptive technology in the COVID-19 era with focused attention to addressing disparities faced by some can reshape health care, especially for rural patients and patients with multiple comorbidities.

The Institute for Healthcare Improvement’s new quadruple aim to optimize health system performance includes improving population health, lowering costs, and improving patient experience (25). The fourth aim is often cited as finding joy in work or elevating health equity (25). These aims may be achieved through a more robust inclusion of telehealth. However, a critical factor for success requires thoughtful supportive interventions to ameliorate reported disparities in telehealth adoption. In the COVID-19 era, informal conversations with health care providers about telemedicine, from primary care to oncology to endocrinology, suggest mixed reactions to virtual visits through telemedicine. Some providers have reverted back to the old ways whereas others have embraced this change. 

Further exploration could identify factors, including barriers, associated with use of telehealth from both the health care provider’s and the patient’s perspective. As long as the CMS policy change for reimbursement remains, a telemedicine visit can be an option between patient and health care provider, and therefore by default create an environment of patient-centered care.

The pandemic crisis has tapped into America’s strengths — our ability to summon unity and collective confidence when facing a nationwide challenge. For telehealth, many of the restrictions have been lifted, namely HIPAA compliance, licensing restrictions, and reimbursement differences by type of visit, with the hope that these will be permanently lifted. Although telemedicine has been integrated into daily clinical practice in responding to the public health emergency, barriers to telemedicine and issues surrounding associated health disparities should not be neglected. Telehealth alone is not a panacea for better health care, and it behooves researchers, providers, and educators to explore creative solutions for optimal health care for all, particularly among vulnerable populations. Undoubtedly, a concerted effort by government agencies, organizations, and community volunteers will be needed to ensure effective access to improved health care, both for high-technology and low-technology solutions.
